# Recovery Observations from Alkali, Nanoparticles and Polymer Flooding as Combined Processes

**DOI:** 10.3390/polym14030603

**Published:** 2022-02-03

**Authors:** Rafael E. Hincapie, Ante Borovina, Elisabeth Neubauer, Muhammad Tahir, Samhar Saleh, Vladislav Arekhov, Magdalena Biernat, Torsten Clemens

**Affiliations:** 1 OMV Exploration & Production GmbH, OMV Upstream Technology & Innovation, New Technology, TECH Center & Lab, 1020 Vienna, Austria; ante.borovina@omv.com (A.B.); elisabeth.neubauer@omv.com (E.N.); vladislav.arekhov@omv.com (V.A.); magdalena.biernat@omv.com (M.B.); torsten.clemens@omv.com (T.C.); 2 Institute of Subsurface Energy Systems, Clausthal University of Technology, Agricolastr. 10, 38678 Clausthal-Zellerfeld, Germany; muhammadtahir04@hotmail.com; 3 Montanuniversität Leoben, DPE Department Petroleum Engineering, Franz-Josef-Straße 18, 8700 Leoben, Austria; samhar@outlook.de

**Keywords:** polymer flooding, alkali–polymer flooding, EOR nanoparticles, interfacial tension, wettability alteration

## Abstract

We have studied wettability alterations through imbibition/flooding and their synergy with interfacial tension (IFT) for alkalis, nanoparticles and polymers. Thus, the total acid number (TAN) of oil may determine the wetting-state of the reservoir and influence recovery and IFT. Data obtained demonstrate how the oil TAN number (low and high), chemical agent and reservoir mineralogy influence fluid–fluid and rock–fluid interactions. We used a laboratory evaluation workflow that combines complementary assessments such as spontaneous imbibition tests, IFT, contact angle measurements and selected core floods. The workflow evaluates wettability alteration, IFT changes and recovery when injecting alkalis, nanoparticles and polymers, or a combination of them. Dynamics and mechanisms of imbibition were tracked by analyzing the recovery change with the inverse bond number. Three sandstone types (outcrops) were used, which mainly differed in clay content and permeability. Oils with low and high TANs were used, the latter from the potential field pilot 16 TH reservoir in the Matzen field (Austria). We have investigated and identified some of the conditions leading to increases in recovery rates as well as ultimate recovery by the imbibition of alkali, nanoparticle and polymer aqueous phases. This study presents novel data on the synergy of IFT, contact angle Amott imbibition, and core floods for the chemical processes studied.

## 1. Introduction

Enhanced oil recovery (EOR) methods promote fluid–fluid (F–F) and rock–fluid (R–F) interactions due to the injection of external agents not initially present in the reservoir [1,2]. Fluid–fluid interactions define static as well as dynamic interfacial properties at the oil–brine–system interface, considering the possible chemical interactions taking place [3,4,5,6,7,8]. Moreover, rock–fluid interactions could result not only in wettability alterations [8,9,10,11,12,13,14], but also in fine migration, often leading to permeability changes [15,16], and other possible mechanisms related to the transport in porous media. The main contributions of both F–F and R–F on additional oil recovery remain subject to study, especially when hybrid processes are applied/evaluated.

Data in the literature show that oil production from chemical EOR has increased over the last decade. For instance, polymer EOR has been implemented in numerous fields, as reported by Delamaide et al. [17], Zhu [18], Kumar et al. [19], Anand et al. [20] and Sieberer et al. [21]. Nevertheless, only a few chemical floods involving the injection of more complex chemical agent systems such as alkali, surfactants or co-solvents, in addition to polymers, have been applied at field scale, as reported by Watson et al. [22], Yin et al. [23], Pitts et al. [24] and Delamaide [25].

Polymer flooding is a widely used and commercially accepted chemical EOR (cEOR) technology [2,26,27]. Polymers increase the viscosity of the aqueous phase and subsequently improve the displacement efficiency due to their favorable mobility ratio. Generally, most researchers believe that polymers do not contribute to enhancing oil recovery, but assist in increasing the ultimate recovery [28]. Some others propose that polymers can contribute to EOR by improving the sweep efficiency [29,30]. Lately, polymers have been used in synergetic applications with other cEOR techniques such as alkali and/or nanoparticle injection seeking to improve R–F/F–F interactions [18,31,32,33,34,35,36,37]. For instance, added alkali/nanoparticles will trigger R–F/F–F interactions, and polymers are expected to contribute as mobility controllers helping recover additional oil due to its better displacement [26,38,39,40,41,42,43].

Alkali flooding is also a proven technology often applied in combination with polymers or surfactants. Alkalis react with the acidic polar compounds present in oil and promote/enhance the generation of in situ soaps (saponify organic acids) [34,44]. This reaction also leads to decreasing IFT at the oil–brine interface. IFT reduction is the result of F–F interactions, causing an increase in the capillary number, hence reducing the residual oil saturation. Capillary number, which relates viscous and capillary forces, is strongly influenced by the IFT and contact angle (wettability alteration) [45]. The roles of both IFT and wettability alterations have been reported in the literature, not only for alkali, but for a combination of cEOR processes [1,44,46,47,48,49,50,51,52]. However, both remain as continuous topics of research when it comes to understanding and designing cEOR applications.

Nanoparticles (NPs) have recently been in the oil industry’s spotlight, showing good outcomes in multiple scientific fields. Improved EOR processes are an NP application area in upstream business. In a nutshell, NPs are expected to promote R–F interactions, leading to oil mobilization; hence, the additional recovery. Different mechanisms have been proposed to explain NP effects in the reservoir, such as IFT reduction, wettability alteration or disjoining pressure, among others [53]. Much research in recent years has shown the NP effects on interfacial tension (F–F interactions) [46,49,54,55,56,57,58,59,60]. However, wettability alterations through NPs are controlled by various factors, such as NP concentration, hydrophobicity, the nature of the reservoir and the NPs [45].

It is generally accepted that one approach to assess wettability alterations during cEOR processes is by performing laboratory test/measurements. Such evaluations include contact angle, spontaneous and force imbibition as a way to validate or understand the changes taking place. Multiple variables play a key role on the evaluations, such as brine/oil composition, the type of chemicals and rock types. For example, some NPs could be more efficient in light oil, whereas others work better in heavy oil [56,58,59,60]. Therefore, the total acid number (TAN) could be another parameter affecting the R–F/F–F interactions during EOR processes. Some researchers also refer to the saponifiable number (SN), and saponifiable acids, as a more reliable comparative variable [61]. TAN defines the number of polar compounds, whereas SN refers to the amount of those compounds able to become soap. In general, acids are also known as surface active agents, composed of naphthenic acid and asphaltenes. Naphthenic acids (cycloaliphatic carboxylic group) are responsible for the oil-wet state of the reservoir rock [4,62]. Moreover, asphaltenes are reported to influence interfacial properties at the oil–brine interface [7,63,64,65]. Therefore, based on the surface-active agent activity, oils with high TAN/SN ratios are good candidates for the synergistic applications of polymers, alkalis and nanoparticles [44].

Synergistic applications of EOR technologies have been reported in the literature with promising results. A compilation of studies assessing the potential and cost-effective synergies of EOR methods is presented in Table 1. Synergies of alkali, nanoparticle and polymer flooding might be promising for excessive oil recovery due to different contributions of each recovery process. Alkalis promote in situ soap generation when reacting with polar compounds of the oil. The saponification process should lower the interfacial tension and could also influence wettability to some extent. The process could be enhanced by NPs due to their reported effects on wettability alterations and lowering the IFT. Furthermore, the presence of polymers will enhance the oil recovery due to good mobility control (improved displacement). The proposed alkali, nanoparticle and polymer synergy differs from conventional ASP flooding due to the dual activity (IFT reduction and wettability alteration) of NPs compared with surfactants (only IFT reductions). Furthermore, the commercial cost/number of surfactants and their limitations to chemical loss (adsorption) at field scale under reservoir conditions have promoted the potential of NPs instead.

In this study, we investigate the dynamics of wettability alterations by integrating different data sources. The results presented here aim show that the workflow can be used as an efficient screening tool to determine the effectiveness of various substances to increase the oil recovery rate and ultimate recovery. Moreover, the workflow helps to understand the synergistic effects of alkalis, nanoparticles and polymer flooding.

## 2. Overall Approach

**Figure 1:**
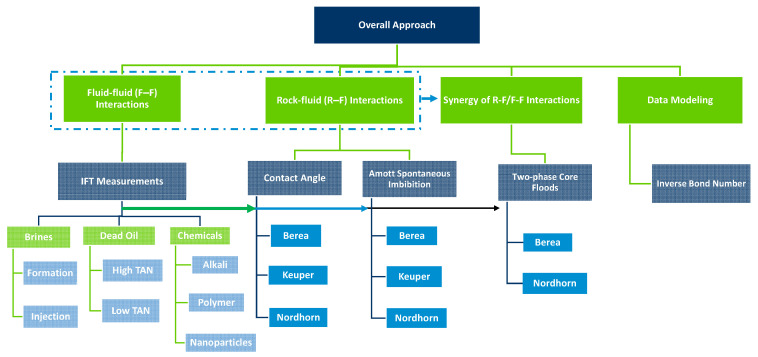
Workflow adopted for the evaluations presented in this paper. The approach incorporates various laboratory evaluations and cross analyses of the data.

To screen injection agents that provide the best synergistic recovery and understand their dynamics using a workflow, the steps presented Figure 1 were undertaken.

## 3. Materials and Methods

### 3.1. Synthetic Brines

For a potential field pilot, it is foreseen to use water produced from the reservoir as injection fluid (softened). Therefore, to mimic the salt content and the buffered behavior of reservoir water, the composition of the injection water (hereafter, test water, TW) included 18.96 g/L NaCl and 1.85 g/L NaHCO_3_ with a pH of 8.93 ± 0.01 and density of 0.994 g/cm^3^, both measured at 23 °C. The formation brine was composed of 19.79 g/L NaCl, 0.40 g/L CaCl_2_·2H_2_O, 0.66 g/L MgCl_2_·6H_2_O and 0.17 g/L NH_4_Cl, with a pH of 8.89 ± 0.01 and a density of 0.995 g/cm3.

### 3.2. Crude Oils

We used oil samples from the 16 TH reservoir of Matzen field (Austria, well Bo-112) and St. Ulrich reservoir (Well StU 65). With the different total acid number (TAN) in both oils, the main aim was to define its impact on the recovery process. The 16 TH oil was more acidic in nature than St Ulrilch oil (TAN = 1.61 mg/KOH/g oil for 16 TH vs. 0.39 mg KOH/g oil for StU); therefore, it was expected to promote more oil wetness. Properties of the crude oil samples are listed in Table 2.

### 3.3. Alkali, Polymer and Nanoparticles

Considering cost and to prevent the formation of silica scales in the production wells due to the dissolution of rock minerals [74,75], Na_2_CO_3_ was investigated here. A known polyacrylamide (HPAM) was used as the polymer: FP3630S, at a concentration of 2000 ppm. For the nanomaterials, four different types were used, mainly silica-coated particles. Further details and specific characterizations of the nanomaterials can be found in Saleh et al. [49] and Neubauer et al. [46,47].

### 3.4. Outcrop Samples

The main properties of the three evaluated sandstone outcrops are presented in Table 3. Berea is a yellowish-grey sandstone that consists of very homogeneous, well-sorted sand comprising, on average, 90% quartz, 4% feldspar, and approximately 6% clays (mainly kaolinite with small contributions of illite and chlorite). Quantities lower than 1% are found for calcite, siderite, and pyrite. Nordhorn is a Bentheimer-like outcrop defined as a fine–medium-grained porous sandstone that consists of 96.6% quartz, 0.9% potassium feldspar and 2.5% kaolinite. Keuper sandstone is a well-sorted arenite containing 97% quartz, 3% clays, <1% plagioclase and calcite, with a particular reddish color given by the iron oxides covering the mineral grains. Further information on the outcrops used in this study can be found in our previous publications: Saleh et al. [49]; Arekhov et al. [1]; and Neubauer et al. [46]. In order to assure consistent rock surface properties, new core plugs were used for each measurement.

### 3.5. Interfacial Tension (IFT) Evaluations

Interfacial tension (IFT) evaluations were performed between different EOR fluids and high-/low-TAN oil in order to investigate the effect. The IFT was measured using a spinning drop tensiometer (SDT) manufactured by Krüss GmbH (Hamburg, Germany). All measurements were conducted at reservoir temperature (60 °C) using a rotational speed of 7000 rpm throughout the 300 min testing time, with a time interval of 20 s between readings.

### 3.6. Amott Spontaneous Imbibition Experiments

Amott spontaneous imbibition experiments were performed using Amott cells, with the oil-saturated cores submerged in the displacing fluid. Oil production was tracked over time by recording the volumes in the pipes at the top of the cells. Experiments were performed at 60 °C and in duplicates or triplicates. Notably, spontaneous imbibition data have not been corrected for oil thermal expansion. Cores were stored for 3 weeks in an oven at 60 °C after being saturated with oil for aging purposes.

### 3.7. Contact Angle Experiments

Contact angle experiments were performed with selected samples using a low-pressure and high-temperature view cell 2 bar (LPCA 202011) in a setup designed by HOT Microfluidics GmbH (Goslar, Germany). Prior to the measurements, cleaned and polished core plugs were aged at atmospheric pressure and at 60 °C for six weeks. A Labotron (LDP 4) pump (Labtron Equipment Ltd. Surrey, UK) was connected to the inlet of the cell to inject the aqueous phase. Pressure exertion occurred at the bottom of the view cell. A capillary was connected to the inlet which dispensed oil for analysis of the contact angle. The cell was exposed to a light source to ensure a high-contrast image. The camera constantly recorded the gathered images from the other side of the cell. Additional information of the setup and testing conditions were presented by Arekhov et al. [1].

### 3.8. Two-Phase Core Floods

Details on the experimental setup are presented in Schumi et al. [44]. Cores were scanned for inhomogeneities and then filled with the TW brine. Cores were stored for 3 weeks in an oven at 60 °C after being saturated with 16 TH oil for aging purposes. The core was mounted vertically and flooded from bottom to top. The injection rate was set to 2 mL/h, corresponding to a frontal velocity of 1 ft/d. Overall, two pore volumes (PVs) of the EOR slug were injected in each experiment. Notably, we compared the selected experiments, because not all were covered in this section. First, cores were flooded with around 1.5 PV of water (brine) until no additional oil was produced. Then, the injection rate was increased to 10 cm^3^/h to ensure that limited mobile oil was left in the core.

### 3.9. Inverse Bond Number Modeling

After spontaneous imbibition tests, data were interpreted using bond number calculations, as described in Schechter et al. [48]. The evaluation uses core and fluid properties in order to delineate the main imbibition mechanisms: capillary or gravity.

$$N_{B}^{-1}=C\frac{\sigma \sqrt{\Phi /k}}{\Delta \rho gH}$$

where *N_B_*^−1^ is the inverse bond number (dimensionless), *C* is a constant (0.4 for the capillary tube model), *σ* is the interfacial tension (N/m), Φ is the porosity (fraction), *k* is the permeability (m^2^), Δ*ρ* is the phase density difference (kg/m^3^), *g* is gravity (m/s^2^), and *H* is the core height in the Amott cell (m).

### 3.10. Post Spontaneous Amott Imbibition Test—Rock Property Evaluation

Dean–Stark and Soxhlet extractions were used to clean the cores. The water and oil were removed by toluene in Dean–Stark apparatus, followed by Soxhlet extraction with methanol to remove salts. Subsequently measurements of porosity and permeability to gas were performed.

## 4. Results and Discussion

### 4.1. Interfacial Tension (IFT) Evaluations

In Table 4, we present a comparison of the IFT values obtained for the fluids discussed in this study. Data are shown comparing high- and low-TAN oil IFTs at two specific reference points. Initial IFT refers to the initial measurement period between elapse times of 10 and 20 min (square root = ~3.5). Equilibrium IFT refers to the value after sufficient interaction time and the value is no longer changing mostly found for a period around 900 min (square root = ~30). In agreement with AlGeer et al. [76], for some cases, a distinct variation is observed between initial and equilibrium IFT values. We attribute the values obtained for the baseline brine to the effect of the buffer capacity of NaHCO_3_. The obtained values are lower than those typically reported for oil–brine systems (~20 mN/m). An overall observation for both oil cases is that the initial and equilibrium IFT barely changed for the three fluids. This is the case for baseline (brine, TW), nanoparticles only and polymers, all dissolved in test water (TW). As reported by Saleh et al. [49], nanomaterials as standalone EOR agents do not lead to remarkable changes in IFT in high-/low-TAN oil. Similarly, as reported by Yin et al. [23], and Arekhov et al. [1], the polymer had a minimal effect on IFT.

For the high-TAN oil, the effect of the alkali solutions on IFT was clear. Alkali (3000 ppm) in TW achieved a 3.7-fold IFT reduction comparing the initial and equilibrium values, whereas for alkali (7000 ppm) in TW, the IFT was reduced 12-fold. When nanoparticles were used together with alkali and NPs with 3000 ppm Na_2_CO_3_ in TW, the reduction was almost threefold. The IFT reduction is attributed to the adsorption of particles onto the oil–water surface, as reported in the literature (e.g., by Saleh et al. [49], Kamal et al. [53], Moradi et al. [58] and Ershadi et al. [54]). The highest reductions were observed with the combination of alkali (7000 ppm Na_2_CO_3_) and polymer (SNF 3630 S) in TW.

For the low-TAN oil, it was observed that small changes took place when the EOR solutions came into contact with the oil. The main explanation is the lack of saponifiable acids in the oil able to generate soaps. In most cases, the initial IFT was lower than equilibrium, with a variation of 0.7-fold. Although for nanoparticles together with alkali (3000 ppm) a 1.27-fold decrease was observed, we considered it a neglectable change.

Conclusively, NPs with alkali solution (3000 ppm) yielded the lowest range of initial and equilibrium IFT in both cases, low-TAN, and high-TAN oil. This lower IFT response could be due to the combined chemical interactions of NPs as well as the alkali.

### 4.2. Contact Angle Observations

An overall comparison is presented in Table 1 for the selected samples. For the base case (brine—TW) of Berea and Nordhorn, we observed that the rock remained water-wet despite the aging. The possible reasons could be limited amount of clay in both rock samples as well as the 100% oil saturation of core plugs. The existing literature supports the hypothesis that a pH value ≤5.5 of formation brine develops a negatively charged rock surface in sandstone (as shown by Tahir et al. [77]). Moreover, the presence of divalent cations in the formation brine helps to build a bridge between the negatively charged rock surface and polar oil compounds, resulting in alterations in the wettability (oil-wet) during the aging process. However, Keuper shifted to oil-wet in the baseline experiments. A significant amount of clay and the presence of iron oxides in Keuper made this wettability shift possible; even without the formation of brine (lower pH value and divalent cations). Conclusively, the aging process of Keuper plugs altered the rock surface, which changed from hydrophilic to hydrophobic, resulting in polar oil compounds attaching to the rock surface. Wettability alterations of the Berea and Bentheimer sandstone core plugs through a two- or three-week aging process have also been established by many researchers (such as Tahir et al. [8]), but with the condition of initial brine saturation (divalent cation and lower pH value). Similarly, we suspect that aging the core plugs in this study changed the wettability to mixed-wet or oil-wet. This is especially the case for Keuper core plugs, as reported by Arekhov et al. [1].

Interestingly, adding chemicals to TW brine (NPs only, alkali only, NPs with alkali or alkali with polymer) showed promising results, changing the wettability of Keuper plugs from oil-wet to water-wet. From Table 5, with the baseline—brine (TW)—the aged Keuper core plug exhibited strong hydrophobic characteristics with a measured contact angle of 149°. However, the contact angle was decreased for all added chemicals to ≈55°, but significantly for NPs, to 31.50°, confirming the change in rock surface to hydrophilic. Hence, wettability alterations could induce polar oil compound detachment and can be seen as additional oil recovery from added chemicals. However, polymers alone cannot develop rock–fluid interactions with a focus on wettability alterations; hence, contact angle measurements with polymer solutions alone were not performed in this study.

From Table 5, we can infer that Keuper core plugs could be the potential core plugs for synergetic EOR mechanisms of IFT reductions, as well as wettability alterations from oil-wet to water-wet system. In contrast, IFT reduction is the main additional recovery mechanism for Berea and Nordhorn, because contact angle measurements confirmed the water-wet condition of both plugs after the aging process. However, the aging process was performed in the absence of any brine formation for these core plugs (100% oil saturation), and the contact angle results differed in the presence of initial brine saturation.

### 4.3. Amott Spontaneous Imbibition Experiments

**Figure 2:**
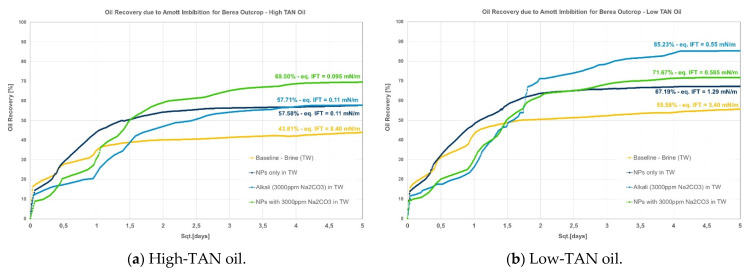
Amott spontaneous imbibition data for evaluations performed in Berea outcrops. (**a**) Recoveries obtained for the high-TAN oil; (**b**) recoveries obtained for the low-TAN oil.

**Figure 3:**
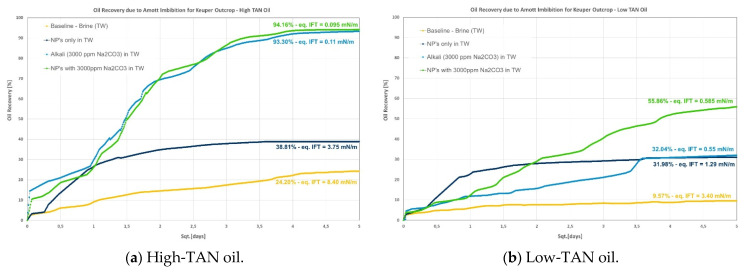
Amott spontaneous imbibition data for evaluations performed in Keuper outcrops. (**a**) Recoveries obtained for the high-TAN oil; (**b**) recoveries obtained for the low-TAN oil.

**Figure 4:**
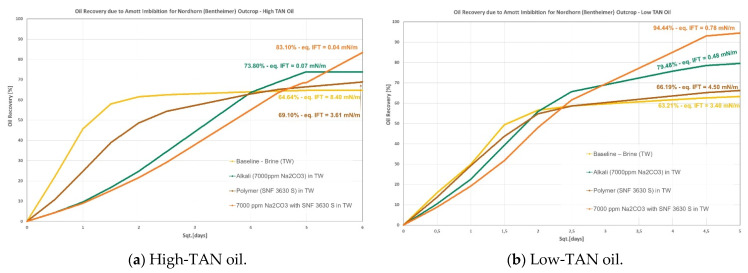
Amott spontaneous imbibition data for evaluations performed in Nordhorn outcrops. (**a**) Recoveries obtained for the high-TAN oil; (**b**) recoveries obtained for the low-TAN oil.

Data are presented per rock type and oil; Figure 2, Figure 3 and Figure 4 show graphical representations of the recoveries obtained, whereas Table 6 and Table 7 report comparisons of recovered oil and incremental values per oil and rock type. Different observations can be pointed out:

- Berea: it was observed that combining nanomaterials with alkali leads to the highest incremental oil, as shown in Figure 2. This is explained by acid–alkali reactions and the generation of in situ soaps, as reported by Saleh et al. [49]. The solution also depicted the lowest IFT of the compared group in Figure 2a for the high-TAN oil. Surprisingly, the contributions of each chemical fluid separately were much lower. In contrast, for the low-TAN oil, it was observed that alkali achieved the highest incremental oil recovery, followed by the nanomaterials and then the alkali;
- Keuper: recoveries in this outcrop clearly presented a difference between high- and low-TAN oil, as shown in Figure 3. The highest recoveries were achieved with the combination of nanomaterials and alkali or by alkali alone. In addition to the IFT, the wettability nature of this outcrop (discussed in the contact angle section) helps explain the observations;
- Nordhorn (Bentheimer): it can be observed from Figure 4 that alkali in combination with a polymer on a similar slug leads to the highest incremental recovery in both oils. Observations were reported by Arekhov et al. [1] for the system with similar IFT and oil. The polymer alone did not lead to any further additional recovery greater than 5%, as shown in Table 6 and Table 7;
- As predicted in the contact angle section, Keuper plugs showed higher additional oil recoveries compared with Berea and Nordhorn core plugs for NPs in alkali chemical systems for both low-TAN and high-TAN oil. This significant additional oil recovery is due to the synergetic effect of NPs and alkali, and resulted in the IFT reduction and wettability alteration from oil-wet to water-wet systems.

### 4.4. Two-Phase Corefloods

A summary of the selected experiments is presented in Table 8, mainly for Berea and Bentheimer (Nordhorn). Results of the contact angle and Amott spontaneous imbibition experiments confirmed the potential of Keuper for ANP flooding; therefore, further investigations focused on the Bentheimer and Berea core plugs to conclude the potential of ANP flood. Only experiments using high-TAN oil were performed in the two-phase corefloods. The highest incremental recoveries were obtained with solution depicting the lowest equilibrium IFT. For evaluations performed using nanomaterials, additional experiments are required to draw better conclusions. Combining nanomaterials with alkali did not lead to substantial additional recovery, as compared with the effects induced by the alkali. In general, incremental recoveries obtained during the Amott imbibition evaluations differed from those in corefloods. We observed a good synergistic approach using the alkali and polymer as one single slug. This is in line with the results extensively reported by Schumi et al. [44].

Furthermore, comparing the data presented in Table 6 and Table 8, Table 9 could be generated. We compared Amott imbibition data with those obtained from coreflooding in high-TAN oil. In the case of Berea, the incremental recoveries from alkali and nanoparticles combined with alkali are similar. Nanomaterials alone lead to different incremental recoveries, which appear to be explained by the nature of the reaction. Some studies report the use of huff-and-puff nanomaterial applications rather than EOR displacement. Interestingly, data obtained for the Nordhorn (Bentheimer) outcrops are very much comparable.

By observing the changes in recovery from Figure 2 to Figure 4, one could argue that there are significant changes. The spontaneous imbibition tests demonstrated the significant impact of the various chemical cocktails on oil recovery. We used Amott cells to assess which chemical cocktail has the potential to significantly improve oil recovery by changing the interfacial tension and wettability. The two-phase tests confirm the selection of the chemical cocktail based on the Amott cells, as presented in Table 9. We performed bump-flooding at the end of the waterflooding; hence, the base case waterflooding was close to residual oil saturation. The incremental oil and remaining oil saturation accordingly were close to residual oil saturation. Regarding water saturation, the impact of the chemical cocktails was not fully tested. This is because they would be injected into an already water-flooded reservoir.

In addition, the role of capillary pressure was explored in this study. The Amott cell tests showed the interplay of capillary pressure and gravity. In terms of capillary pressure, there was wettability concerning real crude oil and interfacial tension. We observed the most significant changes in using chemical agents in oil-wet-prone Keuper core wettability changes, as opposed to Berea and Nordhorn cores. These mostly exhibited water-wet behavior; therefore, their recoveries and recovery rates were mostly influenced by the interplay between capillary pressure (manipulated by changing the IFT) and gravity forces (kept mostly constant for all experiments). Therefore, we manipulated the capillary pressure and imbibition rate by changing the IFT and wettability.

### 4.5. Data Modeling

**Figure 5:**
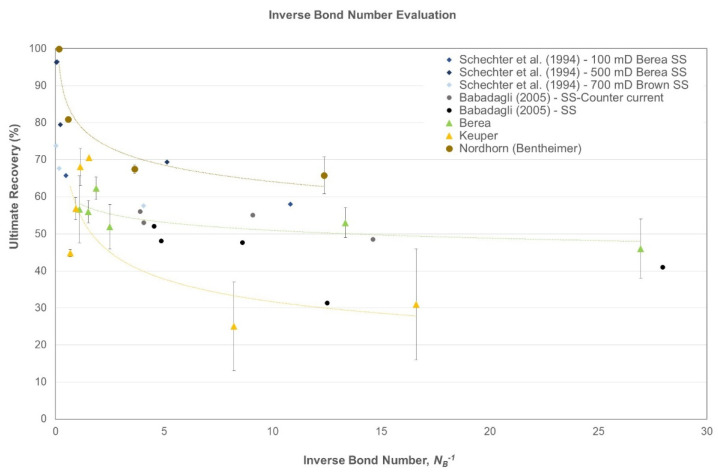
Ultimate recovery vs. inverse bond number as a modeling approach for the data presented in this study.

Figure 5 shows the inverse bond number plotted against ultimate recovery for data for the evaluations performed in this study. Data are compared with those presented by Schechter et al. [48] and Babadagli [52] for sandstone samples. As an overall observation, the inverse bond number (*NB-1*) decreases with the decreasing IFT of the imbibing fluid. In that case, gravity drive contributes to the recovery. Thus, some observations can be identified for each outcrop samples type:

- Berea: This outcrop exhibited general water-wet behavior and the data showed more consistency, even for the lower inverse bond numbers. As reported by Saleh et al. [49] and Neubauer et al. [46], nanomaterial usage indicates a change in wettability as one of the EOR mechanisms. However, the inverse bond number evaluation revealed that a significant amount of additional recovery results from improving the gravity drive by lowering the inverse bond number;
- Keuper: Data presented here fall within the general trend; the data tend to scatter at lower inverse bond numbers for oil-wet Keuper cores. We attribute this behavior to the change in wettability along with lowering of IFT. Moreover, we observed that once inverse bond number increased for baseline experiments, oil recovery dropped, which is in agreement with Schechter et al. [48]. The high standard deviation is attributed to Keuper core heterogeneity;
- Nordhorn (Bentheimer): The curve depicts the highest recovery values for this outcrop type. The general trend is followed, because once the inverse bond number decreases, the ultimate recovery drops.

### 4.6. Porosity and Permeability Changes

A comparison of the permeability and porosity before and after imbibition tests is shown in Table 10. Data are presented for the experiments performed in high-TAN oil mainly because the results for low-TAN oil were similar. A minimal reduction was observed after imbibition with the fluids tested here. In the case of nanomaterials, we assume that possible nanoparticle deposition is not linked to porosity reductions in the cases presented here.

Permeability data also showed some minimal reduction with the tested fluids. Neubauer et al. [46] reported that permeability reduction is more pronounced in Berea outcrop cores as opposed to Keuper cores. Overall, we preliminary conclude that permeability reductions are not associated with the tested nanoparticles present in solution or other fluids. Preliminary analyses such as scanning electron microscopy (SEM) do not depict strong changes.

Notably, we have also investigated the possible effects in the case of nanomaterials in single-phase environment. Arguably, the nanoparticles used here could cause blockages or permeability reductions due to sorption. In our study, Scheurer et al. [78], core flood experiments showed that the injected nanofluids did not have a sufficient effect on permeability.

## 5. Summary and Conclusions

We have identified the conditions leading to increases in recovery rates as well as ultimate recovery by the imbibition of alkali, nanoparticles and polymer aqueous phases. The data obtained demonstrate how the oil TAN number (low and high TAN number) and type of chemical agent influence fluid–fluid and rock–fluid interactions.

The use of alkali or alkali together with nanomaterial in high-TAN oil resulted in a low-equilibrium IFT. It appears that alkali alone falls short of mobilizing trapped low-TAN oil in Keuper cases. Alkali–polymer is efficient in wettability alterations of oil-wet core plugs towards water-wet states for high-TAN oil. The investigated nanomaterials managed to restore a water-wet state in cores with high clay along with improving the gravity-driven flow.

When comparing porosity and permeability before and after imbibition, a slight reduction was observed after imbibition with brine and nanomaterials. We preliminary conclude that permeability reduction is not associated with the tested nanoparticles present in solution. We observed evidence of changes in the imbibition mechanism from counter-current (capillary driven/high inverse bond number) to co-current (gravity-driven/low inverse bond number) for nanoparticles/alkali. The calculated inverse bond number correlates with the ultimate recovery, with a larger inverse bond number leading to lower ultimate recovery.

Overall, we have presented novel data on the synergy of IFT, contact angles, Amott imbibition and coreflooding for the chemical processes studied. We have leveraged from complementary laboratory techniques to define a comprehensive workflow that develops the understanding of wettability-alterations when injecting alkali, nanoparticles and polymers, or a combination thereof. The obtained results show that the workflow can be used as an efficient screening tool to determine the effectiveness of various substances to increase the oil recovery rate and ultimate recovery.

## Figures and Tables

**Table 1:** Selected literature and evaluations of the synergy of alkali/nanoparticles/polymer as cost-effective EOR applications.

Synergy	Reported Recovery Mechanisms	Interaction	Media	Main Focus	Ref.
NPs, P, A	Increase in viscosity + reduction in IFT	R–F and F–F	Micromodel	Contact angle (CA, polymer adsorption	[66]
NPs, P	Increase in polymer viscosity, low polymer adsorption, homogenous NPs dispersion	R–F and F–F	Water-wet micromodels	IFT, CA and nano size distribution	[67]
NPs, P	Higher polymer viscoelasticity, low polymer retention and capillary forces reduction	R–F and F–F	Sandpacks	IFT, contact angle, RRF, viscosity, and relative permeability curves	[68]
A, P, S, NPs, F	Review summary of IFT, polymer adsorption, viscoelasticity, mobility control, wettability alteration, emulsion stabilization,	R–F and F–F	Laboratory to field applications	Review paper covering technical challenges and possible remediations, field projects.	[69]
A, P	IFT reduction and wettability alteration	R–F & F–F	Sandstone cores (oil-wet, water-wet)	Spontaneous imbibition, IFT, contact angle, oil TAN value	[1]
NPs-P, NPs-S, NPs-S-P	Review on IFT reduction & wettability alteration	R–F and F–F	Laboratory to field applications	Review paper covering nanotechnology applications in chemical EOR	[70]
NPs, P, S	IFT reduction, improved rheological properties and wettability alteration	R–F and F–F	Laboratory to field applications	Review on nanotechnology in EOR, challenges, and future research	[71]
NPs, P	Improving viscosity and thermal stability of HPAM polymer	NA	Laboratory to field applications	Review on nanotechnology for improving viscosity and stability	[72]
NPs, A	IFT reduction and wettability alteration	R–F and F–F	Sandstone core plugs	Spontaneous imbibition, IFT tests and phase behavior	[49]
NPs-P	IFT reduction, in situ emulsion generation, microscopic flow and wettability alteration	R–F and F–F	Neutral-wet core plugs	Flooding experiments, IFT, imbibition tests focusing on pressure and recovery	[73]
A, NPs, P	NPs as emulsion stabilizers in AP. IFT and phase behavior. Wettability alteration.	R–F and F–F	Sandstone outcrops	Flooding experiments, IFT, imbibition tests focusing on pressure and recovery	[46,47]

Key: NPs = nanoparticles; P = polymer; A = alkali; AP = alkali–polymer; S = surfactant; F = foam; NPs-P = polymer-coated nanoparticles; NPs-S = surfactant-coated nanoparticles; R–F = rock–fluid; F–F = fluid–fluid.

**Table 2:** Composition of crude oils used in this study.

Property	High TAN	Low TAN
Reservoir/Well	16 TH/Bockfliess 112	St. Ulrich/St.U. 65
TVD top [m]	1622	1060
TAN [mg KOH/g]	1.61	0.39
Saturates [%]	39	55
Aromatics [%]	20	25.6
Resins [%]	39	18.6
Asphaltene [%]	2	0.8
Saponifiable Acids [µmol/g]	26	n.m.
µ @ 60 °C [mPa.s]	11.9	6
ρ @ 20 °C/60 °C [g/cm^3^]	0.917/0.884	0.866/0.842

n.m. = not measured.

**Table 3:** Overall core and saturation data for the outcrop samples used in this study.

Parameter	Units	Berea ^1^		Keuper ^2^		Nordhorn (Bentheimer) ^3^	
		Mean	SD *	Mean	SD *	Mean	SD *
Length	cm	6.97	0.02	8.12	0.09	8.01	0.11
Diameter		2.96	0.01	2.98	0.01	2.96	0.01
Bulk Volume	cm^3^	47.76	0.26	55.76	0.73	54.42	0.87
Pore Volume		10.77	0.19	12.75	0.22	13.07	0.26
Grain Volume	kg/cm^3^	37.00	0.31	42.98	0.67	41.36	0.70
Porosity	%	22.60	0.40	23.30	0.80	23.96	0.35
*N*_2_ permeability (*k_g_*)	mD	447.60	37.40	1425.20	349.60	2313.02	162.10
Water (Test Water) permeability (*k_w_*)		223.90	17.90	890.00	193.90	1501.00	190.12
Irreducible water saturation	%	24.00	8.00	21.40	7.90	25.60	4.00

^1^ Data from 68 core plugs. ^2^ Data from 41 core plugs. ^3^ Data from 26 core plugs. SD * = standard deviation.

**Table 4:** Summary of IFT values of various oil/brine systems in this study.

Fluid	Viscosity [mPa.s] 60 °C, 7.984 s^−1^	High-TAN Oil, [mN/m]				Low-TAN Oil, [mN/m]			
		Initial IFT		Equilibrium IFT		Initial IFT		Equilibrium IFT	
		Mean	SD *	Mean	SD *	Mean	SD *	Mean	SD *
Baseline—Brine (TW)	0.571	7.84	0.43	8.40	0.50	4.31	0.62	3.40	0.56
NPs only in TW ^1^	5.325	3.67	0.20	3.75	0.20	2.02	0.18	1.29	0.01
Alkali (3000 ppm Na_2_CO_3_) in TW	0.559	0.41	0.62	0.11	0.07	0.70	0.28	0.55	0.15
Alkali (7000 ppm Na_2_CO_3_) in TW	0.601	0.87	0.19	0.07	0.01	0.34	0.11	0.48	0.18
NPs with 3000 ppm Na_2_CO_3_ in TW	7.254	0.27	0.02	0.095	0.01	0.775	0.04	0.585	0.01
Polymer (SNF 3630 S) in TW	19.536	3.31	0.25	3.61	0.36	4.03	0.37	4.50	0.22
7000 ppm Na_2_CO_3_ with SNF 3630 S in TW	18.054	2.41	0.55	0.04	0.01	0.48	0.09	0.78	0.18

^1^ Average of three nanomaterials at 0.1% wt. and 15 measurements each. SD * = standard deviation.

**Table 5:** Summary of selected contact angle data expressed in degrees [°] measured at 60 °C. Data were measured at 300 min observation for the fluid samples used in this study.

Fluid	Berea		Keuper		Nordhorn (Bentheimer)	
	High TAN	Low TAN	High TAN	Low TAN	High TAN	Low TAN
Baseline—Brine (TW)	30.00	n.m.	149.00	60.70	58.70	60.70
NPs only in TW^1^	33.50	n.m.	31.50	n.m.	n.m.	n.m.
Alkali (3000 ppm Na_2_CO_3_) in TW	35.01	n.m.	55.76	n.m.	n.m.	n.m.
Alkali (7000 ppm Na_2_CO_3_) in TW	n.m.	n.m.	54.20	47.80	57.20	42.70
NPs with 3000 ppm Na_2_CO_3_ in TW	33.20	n.m.	46.02	n.m.	n.m.	n.m.
7000 ppm Na_2_CO_3_ with SNF 3630 S in TW	n.m.	n.m.	56.10	55.90	57.80	59.80

n.m. = not measured; ^1^ Average of two nanomaterials.

**Table 6:** Summary of recoveries [%] obtained for high-TAN oil during Amott spontaneous imbibition for the fluid samples used in this study.

Imbibing Fluid	Berea, [%]		Keuper, [%]		Nordhorn (Bentheimer), [%]	
	R.O	I.O.	R.O	I.O.	R.O	I.O.
Baseline—Brine (TW)	43.81	-	24.20	-	64.64	-
NPs only in TW ^1^	57.58	13.77	38.31	14.11	n.m.	n.m.
Alkali (3000 ppm Na_2_CO_3_) in TW	57.71	13.90	93.30	69.10	n.m.	n.m.
Alkali (7000 ppm Na_2_CO_3_) in TW	n.m.	n.m.	n.m.	n.m.	73.80	9.16
NPs with 3000 ppm Na_2_CO_3_ in TW	69.50	25.69	94.16	69.96	n.m.	n.m.
Polymer (SNF 3630 S) in TW	n.m.	n.m.	n.m.	n.m.	69.10	4.46
7000 ppm Na_2_CO_3_ with SNF 3630 S in TW	n.m.	n.m.	n.m.	n.m.	83.10	18.46

n.m. = not measured; ^1^ Average of two nanomaterials; R.O. = recovered oil; I.O. = incremental oil (corrected to brine recovery).

**Table 7:** Summary of recoveries [%] obtained for low-TAN oil during the Amott spontaneous imbibition for the fluid samples used in this study.

Imbibing Fluid	Berea, [%]		Keuper, [%]		Nordhorn (Bentheimer), [%]	
	R.O	I.O.	R.O	I.O.	R.O	I.O.
Baseline—Brine (TW)	55.58	-	9.57	-	63.21	-
NPs only in TW ^1^	67.19	11.61	31.98	22.41	n.m.	n.m.
Alkali (3000 ppm Na_2_CO_3_) in TW	85.23	29.65	32.04	22.47	n.m.	n.m.
Alkali (7000 ppm Na_2_CO_3_) in TW	n.m.	n.m.	n.m.	n.m.	74.48	11.27
NPs with 3000 ppm Na_2_CO_3_ in TW	71.67	16.09	55.86	46.29	n.m.	n.m.
Polymer (SNF 3630 S) in TW	n.m.	n.m.	n.m.	n.m.	66.19	2.98
7000 ppm Na_2_CO_3_ with SNF 3630 S in TW	n.m.	n.m.	n.m.	n.m.	94.44	31.23

n.m. = not measured; ^1^ Average of two nanomaterials; R.O. = recovered oil; I.O. = incremental oil (corrected to brine recovery).

**Table 8:** Summary of selected core flood data for the fluids used in this study. Data shown here are for experiments performed in high-TAN oil only.

Injected Fluid	Equilibrium IFT	Berea		Nordhorn (Bentheimer)	
	High TAN, [mN/m]	Incremental Recovery%	Injected PV	Incremental Recovery%	Injected PV
NPs only in TW ^1^	3.75	3.90	2.00	n.p.	n.p.
Alkali (3000 ppm Na_2_CO_3_) in TW	0.11	14.00	2.00	12.00	1.5
Alkali (7000 ppm Na_2_CO_3_) in TW	0.07	n.p.	n.p.	19.00	1.5
NPs with 3000 ppm Na_2_CO_3_ in TW	0.095	18.00	2.00	n.p.	n.p.
Polymer (SNF 3630 S) in TW	3.61	9.00	2.00	3.00	3.0
7000 ppm Na_2_CO_3_ with SNF 3630 S in TW	0.04	n.p.	n.p.	29.00	2.00

n.p. = not performed; ^1^ Average of two nanomaterials.

**Table 9:** Comparison of selected data between imbibition and coreflooding fluids in this study. Data shown here are for experiments performed with high-TAN oil only.

Injected Fluid	Equilibrium IFT	Berea, Incremental Oil [%]		Nordhorn (Bentheimer), Berea, Incremental Oil [%]	
	High TAN, [mN/m]	Imbibition	Flooding	Imbibition	Flooding
NPs only in TW ^1^	3.75	13.77	3.90	n.m.	n.p.
Alkali (3000 ppm Na_2_CO_3_) in TW	0.11	13.90	14.00	n.m.	12.00
Alkali (7000 ppm Na_2_CO_3_) in TW	0.07	n.m.	n.p.	11.27	19.00
NPs with 3000 ppm Na_2_CO_3_ in TW	0.095	25.69	18.00	n.m.	n.p.
Polymer (SNF 3630 S) in TW	3.61	n.m.	9.00	2.98	3.00
7000 ppm Na_2_CO_3_ with SNF 3630 S in TW	0.04	n.m.	n.p.	31.23	29.00

n.p. = not performed; n.m. = not measured; ^1^ Average of two nanomaterials.

**Table 10:** Summary comparison of porosity and permeability before and after Amott imbibition experiments. Data shown here are for experiments performed in high-TAN oil only.

Imbibing Fluid	Outcrop	Porosity Φ, [%]			Permeability, [mD]		
		Before	After	Diff. (%)	Before	After	Diff. (%)
NPs only in TW ^1^	Berea	22.60	21.34	−1.83	477.62	419.82	−12.14
	Keuper	23.49	23.17	3.57	1381.62	1311.50	−5.17
Alkali (3000 ppm Na_2_CO_3_) in TW	Berea	22.58	22.12	−2.04	399.55	364.44	−8.82
	Keuper	24.09	23.99	−1.10	1238.83	1225.16	−1.10
	Nord. (Bent)	23.30	23.05	−1.07	2343.05	2340.02	−0.15
Alkali (7000 ppm Na_2_CO_3_) in TW	Nord. (Bent)	24.30	24.10	−0.90	2448.62	2389.78	−2.40
NPs with 3000 ppm Na_2_CO_3_ in TW	Berea	22.42	22.23	−0.77	442.05	391.859	−11.32
	Keuper	24.28	23.69	−2.63	1446.37	1320.94	−8.03
Polymer (SNF 3630 S) in TW	Nord. (Bent)	24.19	24.08	0.61	2346.49	2273.49	−3.11
7000 ppm Na_2_CO_3_ with SNF 3630 S in TW	Nord. (Bent)	23.98	24.45	1.95	2310.33	2113.95	−8.50

n.p. = not performed; ^1^ Average of two nanomaterials.

## Data Availability

Not applicable.
